# Automating the Management of Extraspinal Findings in Magnetic Resonance Imaging Spine Studies Using a Privacy-Preserving Large Language Model: Retrospective Validation Study

**DOI:** 10.2196/86251

**Published:** 2026-08-26

**Authors:** Wilson Ong, Gifford Tan, Yong Han Ting, Shuliang Ge, Yi Liang Tan, Xi Zhen Low, Wei Chuan Tan, Andrew Makmur, Naomi Wenxin Leow, Muzammil Arif Din Abdul Jabbar, Qai Ven Yap, Shao Jin Ong, Jiong Hao Jonathan Tan, Naresh Kumar, James Thomas Patrick Decourcy Hallinan

**Affiliations:** 1 Department of Diagnostic Imaging, National University Hospital Singapore Singapore; 2 Department of Diagnostic Radiology, Yong Loo Lin School of Medicine, National University of Singapore Singapore Singapore; 3 Artificial Intelligence Innovation Office National University Health System Singapore Singapore; 4 School of Clinical Medicine, University of Cambridge Cambridge United Kingdom; 5 Agency for Science, Technology and Research (A*STAR) Singapore Singapore; 6 Biostatistics Unit, Yong Loo Lin School of Medicine, National University of Singapore Singapore Singapore; 7 National University Spine Institute, Department of Orthopaedic Surgery, National University Health System Singapore Singapore

**Keywords:** large language model, extraspinal findings, referrals, incidental findings, magnetic resonance imaging

## Abstract

**Background:**

Magnetic resonance imaging (MRI) spine studies frequently reveal extraspinal findings (ESFs) that require further evaluation; yet, the current process of manually reviewing radiology reports and navigating electronic medical records (EMRs) is time-consuming, labor-intensive, and prone to human error.

**Objective:**

To address this challenge, we propose using a privacy-preserving large language model (PP-LLM) to automate the identification, classification, and referral assessment of ESFs.

**Methods:**

A retrospective analysis of 405 consecutive MRI spine reports from the National University Hospital database, covering February to June 2024, was conducted. Two independent clinicians reviewed the reports and cross-referenced them with EMRs to identify ESFs from the imaging reports. The CT Extracolonic Findings Reporting and Data System (C-RADS) was adapted to determine the clinical significance of ESFs and whether specialty referral was required. The PP-LLM was designed to extract these findings, differentiate between new and preexisting conditions, classify their clinical significance, and generate appropriate referrals.

**Results:**

A total of 405 MRI spine reports were initially identified. Five reports were excluded because no relevant EMRs were available, leaving 400 MRI reports from 395 patients for analysis. Among 395 patients (male: 48.1%, n=190; female: 51.9%, n=205; age: mean 54.7, SD 16.7, range 17-89 years), 163 (41.3%) had no ESFs and 232 (58.7%) were reported to have had at least one ESF. A total of 401 ESFs were identified, with the most common findings being renal (n=128, 31.9%), gynecological (n=110, 27.4%), and endocrine-related (n=48, 12%). The PP-LLM correctly detected 99.8% (400/401) of all ESFs and correctly identified all clinically urgent findings (3%, 12/401 of the total, for example, aortic dissection). It misclassified 2% (8/401) of cases into lower C-RADS categories, potentially downgrading clinically significant findings (eg, paranasal sinus mucosal thickening), and 1% (4/401) into higher C-RADS categories, upgrading clinically insignificant findings (eg, dependent changes in the lungs). Additionally, it achieved 98.5% accuracy in distinguishing new from preexisting findings and 96.3% accuracy in assigning the correct specialty referral decision (whether referral is needed, and correct subspecialty referral suggested). The PP-LLM demonstrated almost perfect agreement with the reference standard (Gwet κ=0.959, 95% CI 0.937-0.981), comparable to two human readers (reader 1: κ=0.943; reader 2: κ=0.934), with no statistically significant difference in C-RADS classification accuracy. Notably, it completed the analysis of each report in less than 5 seconds.

**Conclusions:**

The PP-LLM demonstrated high accuracy and efficiency in automating the identification and classification of ESFs in MRI spine reports. By integrating this AI-driven automation into clinical workflows, this technology has the potential to enhance efficiency, reduce clinician administrative burden, ensure timely specialist referrals, and improve patient care.

## Introduction

In current medical practice, the efficient management of large volumes of patient data is essential, particularly in specialized fields such as spine surgery. At many spine clinics, hundreds of patients undergo magnetic resonance imaging (MRI) spine examinations, each yielding a detailed radiology report that includes both spinal findings and significant extraspinal abnormalities [1]. These extraspinal findings (ESFs) may encompass a wide range of clinically relevant conditions, including renal cysts and masses, aortic pathologies such as dissections or aneurysms, uterine fibroids or masses, adnexal lesions, lymphadenopathy, lung nodules or masses, and thyroid abnormalities [2,3]. The breadth and depth of these reports demand a meticulous review process to ensure that critical findings are identified and appropriately acted upon in a timely fashion [4]. This task is further complicated by substantial variability in reporting styles, the extensive use of domain-specific terminology, and frequent reliance on assumed clinical knowledge, which makes consistent interpretation challenging [5].

Typically, the spine surgeon must assess these ESFs against the patient's electronic medical records (EMRs) to determine whether they are preexisting conditions or new developments. If an ESF is identified as preexisting, no further action is required. However, if the finding is new, it may necessitate a referral to the appropriate specialist for further evaluation and management. This manual process of cross-referencing findings with patient history is time-consuming and adds to the already significant workload in the clinic [6].

To address these challenges, we propose the implementation of a privacy-preserving large language model (PP-LLM) to automate and streamline this workflow. Large language models (LLMs) have been shown to support a range of health care tasks in the literature, including triage in emergency settings [7], automated clinical trial screening [8], and radiology request form enhancement and auto-protocoling [9]. Our proposed method of using PP-LLM could automate the identification and triage of ESFs by scanning radiology reports, cross-referencing them with EMRs, and generating referral recommendations based on standardized criteria. By improving the consistency and speed of referral decisions while maintaining patient confidentiality, the PP-LLM offers a promising solution to streamline care delivery and reduce the administrative burden for spine surgeons and other clinicians.

In this study, we will evaluate the performance of the institutional PP-LLM in reviewing ESFs from MRI spine reports at our institution. The PP-LLM is designed to perform 4 key tasks: first, to detect and list ESFs documented in the radiology reports; second, to determine whether these findings are preexisting or new by cross-referencing with the patient's EMR; third, to classify their clinical significance; and fourth, to generate automatic referral letters to the relevant specialists for any new findings identified. The outputs of the PP-LLM will be compared against a reference standard provided by two experienced clinicians to assess its accuracy in identifying and classifying ESFs. This evaluation forms the basis for a broader effort to fully implement the PP-LLM into routine clinical care.

## Methods

### Ethical Considerations

This study was granted a waiver of informed consent by the institutional review board under the domain-specific review board, based on the minimal-risk nature of the project and its compliance with ethical standards. The waiver was granted due to the retrospective use of clinical data collected as part of routine care, in accordance with institutional policies. All study data were handled within a secure institutional environment and were deidentified prior to analysis. Access to EMR data and imaging reports was restricted to authorized study personnel in compliance with institutional data governance and privacy protection policies. The institutional PP-LLM was deployed within a secure infrastructure and processed only authorized clinical information. No participants received financial compensation, as the study involved retrospective analysis of existing clinical data. No identifiable patient information is included in the manuscript or supplementary materials, and all data presented have been anonymized in accordance with institutional and journal requirements.

### Clinical Data Collection

A retrospective analysis of 405 consecutive MRI spine reports from the National University Hospital database, covering February to June 2024, was conducted. Each report documented findings in the spine and noted any significant ESFs. The last relevant clinical entries from the EMR of these patients were also extracted to determine the clinical relevance and need for further action regarding the ESFs. Inclusion criteria were patients with clinical entries accessible on the EMR to allow the relevant clinical history to be analyzed. Patients without any accessible clinical entries in the EMR were excluded from analysis. MRI spine examinations are typically ordered following at least one prior clinical encounter, and therefore at least one corresponding EMR entry would ordinarily be expected. In 5 cases, no clinical documentation was available despite imaging having been performed, introducing uncertainty regarding whether these represented data extraction limitations or atypical clinical workflow scenarios in which the patient had not yet been formally assessed by the ordering clinician. Because the novelty classification of ESFs required comparison with prior documented clinical history, these cases were excluded to avoid ambiguity in determining whether the findings were new or preexisting.

**Figure 1:**
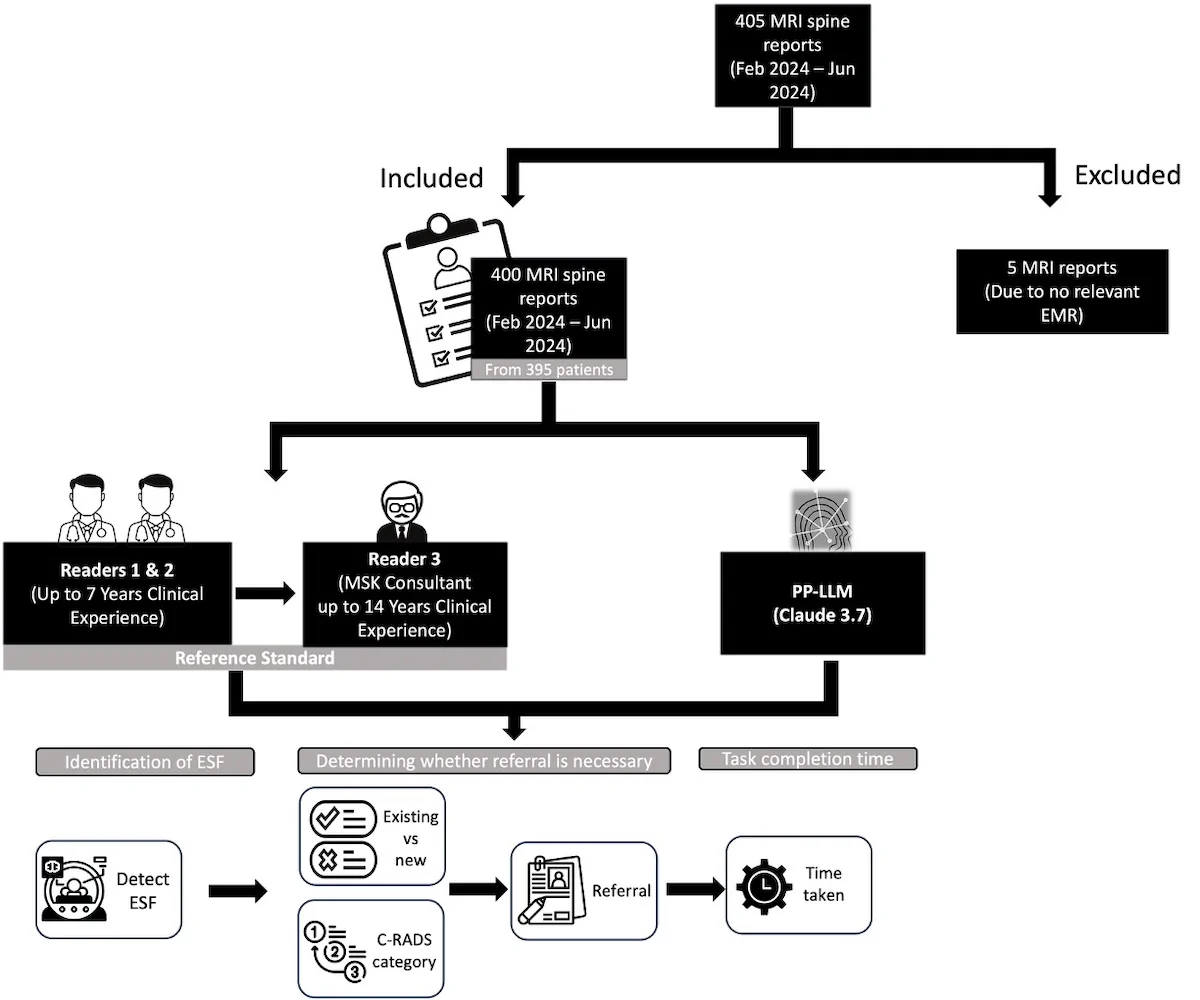
Flowchart of the study design. A total of 400 out of 405 MRI reports (5 excluded due to no relevant EMR) from 395 patients were included for analysis. Original MRI reports were first reviewed by two clinicians to identify ESF. For each ESF, reviewers (readers 1 and 2) determined whether the finding was previously known or new using EMR, assessed clinical significance using a modified C-RADS (E1-E4), and evaluated the need for subspecialty referral (for previously unknown ESF graded E3 or E4). Discrepancies were resolved by consensus with a third independent reviewer—reader 3 (senior clinician–musculoskeletal radiologist)—to establish the reference standard. The performance of a secure LLM, Claude version 3.7 Sonnet (Anthropic), was then evaluated against this standard. The model was also required to perform the same four-layered analysis: (1) identify ESF, (2) classify novelty, (3) triage clinical significance, and (4) assess referral necessity. C-RADS: CT Extracolonic Findings Reporting and Data System; EMR: electronic medical record; ESF: extraspinal finding; MRI: magnetic resonance imaging; MSK: musculoskeletal radiologist; PP-LLM: privacy-preserving large language model.

Each MRI spine report was independently reviewed by two clinicians (readers 1 and 2, each with up to 7 years of clinical experience) to identify ESFs (Figure 1). Identified findings were cross-referenced against the most recent relevant clinical entry in the EMR, including documented past medical history within a predefined 6-month look-back window prior to the MRI examination, to determine whether ESFs represented new observations or previously known conditions requiring further evaluation. This timeframe was selected to capture clinically relevant recent diagnoses and management decisions while maintaining a practical and reproducible review window. The same EMR look-back window and contextual inputs were provided to both human reviewers and the PP-LLM to ensure methodological consistency when determining whether ESFs were new or preexisting.

For ESFs that were already known, no further action was required. For ESFs that were new observations, these were further classified based on their clinical significance and whether subspecialty referral was required. This manual assessment was performed independently by the two clinicians (readers 1 and 2) to ensure accuracy and reduce potential bias. The results were then compared, and any major discrepancies were resolved by a third independent clinician (reader 3 with up to 14 years of clinical experience).

Because the reference standard was derived through consensus adjudication involving the primary readers and a senior reviewer, comparisons between individual readers and the reference standard may be subject to incorporation bias. However, consensus adjudication with an independent senior clinician is a commonly accepted approach in radiology reader-performance studies and was used to improve the reliability of the ground truth labels. To classify the clinical significance of each ESF and determine whether subspecialty referral was required, we applied a modified version of the CT Extracolonic Findings Reporting and Data System (C-RADS) [10]. This adapted framework incorporated modifications based on referral and management practices specific to our local university hospital (Multimedia Appendices 1 and 2). In general, anatomic variants were categorized as C-RADS E1 (normal findings or anatomic variants); C-RADS E2 was assigned to clinically unimportant findings that required no further work-up (eg, simple renal cysts and diverticulosis); C-RADS E3 encompassed indeterminate or incompletely characterized findings for which clinical correlation and additional work-up, including subspecialty referral, could be considered if indicated (eg, minimally complex renal cysts); and C-RADS E4 designated potentially important findings requiring further investigation and early communication with the referring physician, in line with established clinical guidelines (eg, solid renal mass and abdominal aortic aneurysm).

For ESFs associated with multiple acceptable subspecialty referral pathways (Multimedia Appendix 2; for example, hepatology/gastroenterology or general surgery for selected hepatic lesions), referral predictions were scored as correct if the model selected any one of the predefined acceptable specialties. This approach was intended to reflect real-world clinical practice, in which referral pathways may vary across institutions or depend on local workflow and clinician preference.

Although the original C-RADS framework was developed for extracolonic findings detected on CT colonography, its clinical-significance categories (E1/E2, E3, and E4) were applied in this study as a generalized risk-stratification framework across ESFs identified on cervical, thoracic, and lumbar spine MRI examinations. This modified institutional adaptation reflects local referral pathways and management priorities for incidental findings across multiple anatomical regions rather than being limited to abdominal or pelvic pathology.

### Secure Institutional LLM Details

A PP-LLM (Claude 3.7 Sonnet; Anthropic) was evaluated within a fixed inference period between January 2025 and May 2025 using a controlled institutional deployment pipeline configured for clinical evaluation workflows. The model was accessed through an institutionally governed deployment interface configured for clinical evaluation workflows rather than unrestricted public API usage. The PP-LLM workflow was implemented within a secure institutional computing environment in which EMR data and radiology reports remained within the hospital network during preprocessing and inference. No protected health information was transmitted outside the institutional clinical environment as part of the study workflow.

Access to EMR data and imaging reports was restricted to authorized study personnel in accordance with institutional cybersecurity policies, role-based access control requirements, and institutional review board approval. Relevant prior clinical entries were retrieved directly from the EMR and provided as structured contextual input to the model through a supervised retrieval pipeline designed to minimize the exposure of unnecessary clinical information. All data were deidentified prior to model input using a combination of institutional digital deidentification tools and manual verification to ensure removal of directly identifiable patient information. No persistent storage of identifiable clinical inputs was performed within the model environment beyond the duration required for inference processing.

The LLM was provided with relevant contextual data from the EMR, including the most recent clinical entry by the referring medical team (including known past medical history) and prior imaging reports within a 6-month look-back period. A formal retrieval-augmented generation (RAG) indexing framework was not implemented. Instead, relevant prior clinical entries were retrieved directly from the EMR and supplied as structured contextual inputs as part of the workflow pipeline. Prompt instructions were iteratively optimized by two clinicians (readers 1 and 2) using an instruction-following, zero-shot chain-of-thought prompt with explicit task decomposition and constrained decision rules. The task scope was restricted to nonspinal findings, and outputs were standardized to C-RADS categories to mitigate hallucination risk and enhance reproducibility. Task decomposition into discrete chain-of-thought logical components improved interpretability, reflected real-world clinical reasoning processes, and facilitated stepwise auditability for internal consistency. Reverse prompt engineering was applied to remove redundant content and minimize ambiguity. Prompt instructions were optimized using 20 practice spine MRI reports and corresponding EMR entries that were separate from the study cohort. The optimized prompt instructed the model to:

1. Extract any findings in the MRI report not related to the spine, but do not use the clinical history for this.
2. For each finding, decide its C-RADS category based on the provided table (Multimedia Appendix 1). Classify them into E1/E2, E3, or E4.
3. Then look at the clinical history to see if it is preexisting.
4. If it is not preexisting, let me know if this requires referral (yes or no).
5. Indicate which subspecialty to refer to.

The 20 practice MRI spine reports and corresponding EMR entries used for prompt optimization were separate from the study cohort and were not included in the final dataset of 400 MRI spine reports analyzed in this study. These cases were used only during the prompt development phase to refine task instructions and improve structured output consistency, thereby avoiding potential data leakage into the evaluation dataset. Within the prompt design, extraction of ESFs was intentionally performed using only the MRI report text to simulate the initial radiology-report interpretation workflow. The clinical history from the EMR was subsequently incorporated in a separate step to determine whether identified findings were preexisting or new and to assess referral necessity. This sequential task structure reflects the intended workflow logic rather than contradictory instructions and mirrors real-world clinical decision-making processes. The evaluated prompt instructed the PP-LLM to identify ESFs, assign modified C-RADS categories, determine whether findings were new or preexisting, assess referral necessity, and indicate the appropriate subspecialty referral. Automated generation of referral letter text was not included within the evaluated prompt and instead represents a proposed downstream clinical implementation following identification of new E3/E4 findings requiring specialist referral.

### MRI Report Task and Analysis

The PP-LLM was then used to automate and perform the following tasks:

1. Identification of ESFs: The PP-LLM was programmed to scan and list all significant ESFs documented in the MRI spine reports.
2. Assigning appropriate C-RADS categories: The PP-LLM was then tasked to classify the ESFs detected to various C-RADS categories (E1 to E4).
3. Determination of referral necessity: The model then cross-referenced these findings with the patient’s medical history recorded in the EMR. The PP-LLM will then assess referral necessity, and if an E3 or E4 ESF is determined to be new, the PP-LLM will prompt a referral necessity and indicate the appropriate subspecialty referral. If the finding was already known, no referral was suggested. Referral to the relevant subspecialty is determined by institutional referral pathways and clinical consensus (eg, renal findings to urology, thyroid findings to endocrinology/ENT).

The total end-to-end automated inference time required for the PP-LLM to complete the full analysis pipeline for a single case is then recorded. This included extraction of ESFs from the MRI report text, cross-referencing with the last relevant EMR entry, determination of whether findings were new or preexisting, assignment of modified C-RADS category, and generation of referral recommendations with the corresponding subspecialty destination when indicated.

Importantly, there are two possible deployment pathways: (1) the referral letter can be generated and placed in a sidebar or dashboard for final clinician review and approval before sending or (2) it can be transmitted directly. In practice, a final human check is expected, given that the patient would need to be consulted, and the referral letter would typically be presented to the clinician in a ready-to-send format to save time. This ensures that clinician oversight is preserved while still reducing administrative workload.

### Results Analysis

Patient demographics and the overall prevalence of ESFs were summarized using descriptive statistics. These were used to characterize the study population and the distribution of ESFs. A reference standard was established through independent manual review of the MRI spine reports by the two human readers (readers 1 and 2). Discrepancies were resolved by consensus with a third senior clinician (reader 3). The interrater agreement between the two primary reviewers, the PP-LLM, and the reference standard for C-RADS classification was evaluated using Gwet κ. Gwet κ was selected over Cohen κ to account for the prevalence and marginal distribution effects that can distort agreement estimates in imbalanced datasets. Agreement strength was interpreted using the Landis and Koch scale: <0 (poor), 0.00-0.20 (slight), 0.21-0.40 (fair), 0.41-0.60 (moderate), 0.61-0.80 (substantial), and 0.81-1.00 (almost perfect agreement). Statistical significance for Gwet κ coefficients was evaluated against the null hypothesis of chance-level agreement (κ=0). These analyses were used to benchmark the consistency of expert reviewers and assess the comparative performance of the PP-LLM.

The performance of the PP-LLM was subsequently assessed against the reference standard in three areas: (1) detection of ESFs, (2) assignment of the appropriate modified C-RADS category (E1-E4), and (3) determination of referral necessity and subspecialty. A decision by the PP-LLM was considered correct when its identification of ESFs, C-RADS classification, and referral recommendation—including subspecialty assignment—matched the consensus determination established by the human reviewers (reference standards).

Performance metrics—including sensitivity, specificity, precision, *F*_1_-score, and overall accuracy—were computed for a descriptive evaluation of model performance relative to the reference standard. Confidence intervals were calculated for Gwet κ coefficients to assess agreement precision. Confidence intervals were not calculated for sensitivity, specificity, or *F*_1_-score because these metrics were used primarily for descriptive benchmarking rather than inferential hypothesis testing. To compare the classification performance of the PP-LLM and human reviewers, a continuity-corrected McNemar test (Edwards correction) was applied to paired binary outcomes (correct versus incorrect classification relative to the reference standard) rather than directly comparing multiclass category assignments. Because both the PP-LLM and human readers were evaluated on the same set of cases against a shared reference standard, the comparisons were inherently paired, and a continuity-corrected McNemar test was used to assess differences in paired proportions between classifiers. This binary reduction approach is consistent with established methodology for reader-performance comparisons involving categorical classification tasks. All performance metrics were calculated using the full reference dataset of 401 ESFs, and findings not detected by the readers were treated as false negatives and were retained in the denominator during performance evaluation. All analyses were conducted using Python version 3.9.12 and IBM SPSS Statistics (version 30).

## Results

### Demographics and Extraspinal Findings

A total of 405 MRI reports were extracted, 5 of which were excluded due to no relevant EMR, and 400 MRI reports were included for analysis. The 400 MRI reports belonged to 395 patients (male: 48.1%, n=190; female: 51.9%, n=205; age: mean 54.7, SD 16.7 years), and most had either no ESFs (41.3%, n=163) or a single finding (31.9%, n=126). Fewer patients had multiple findings: 15.2% (n=60) with 2 ESFs, 7.8% (n=31) with 3 ESFs, 3.3% (n=13) with 4 ESFs, and only 0.5% (n=2) with 5 ESFs (Table 1). In total, 401 ESFs were identified across all patients (Table 2). The numbers reported for readers 1 and 2, and the PP-LLM in Table 2 represent predicted classifications rather than reference standard labels; therefore, small differences between these values and the ground truth totals (352 new and 49 known ESFs) reflect expected classification errors (false positives and false negatives). The most common categories of findings (Multimedia Appendix 2) were renal (31.9%, 128/401), gynecological (27.4%, 110/401), and endocrine-related (12%, 48/401). Using a modified C-RADS classification system, 95 (23.7%) ESFs were categorized as E1/E2 (clinically insignificant), 294 (73.3%) as E3 (clinically indeterminate and requiring further evaluation), and 12 (3%) as E4 (clinically significant and requiring prompt follow-up).

**Table 1:** Patient and spine MRI^a^ study details.

Characteristics			Values
**Patient demographics (n=395)**			
	**Age (years)**		
		Mean (SD)	54.7 (16.7)
		Range	17-89
	**Sex, n (%)**		
		Male	190 (48.1)
		Female	205 (51.9)
**Spine MRI studies (n=400)**			
	**Treatment setting, n (%)**		
		Inpatient	57 (14.2)
		Outpatient	343 (85.8)
	**Spine regions, n (%)**		
		Cervical	51 (12.8)
		Thoracic	14 (3.5)
		Lumbar	273 (68.2)
		Whole spine	62 (15.5)
**Patients with extraspinal findings (ESFs; n=395), n (%)**			
	No ESF		163 (41.3)
	1 ESF		126 (31.9)
	2 ESFs		60 (15.2)
	3 ESFs		31 (7.8)
	4 ESFs		13 (3.3)
	5 ESFs		2 (0.5)

^a^MRI: magnetic resonance imaging.

**Table 2:** Accuracy of ESF^a^ detection, C-RADS^b^ classification, and need for referral of human readers (readers 1 and 2) versus privacy-preserving large language model (PP-LLM).

			Reader 1		Reader 2		PP-LLM
**ESF detected**							
	Total (n=401), n/N (%)	401/401 (100)		399/401 (99.5)		400/401 (99.8)	
**ESF classified, n (%)**							
	C-RADS E1/E2 (n=95, 23.7%)	101 (25.2)		100 (24.9)		98 (24.4)	
	C-RADS E3 (n=294, 73.3%)	288 (71.8)		287 (71.6)		290 (72.3)	
	C-RADS E4 (n=12, 3%)	12 (3.0)		12 (3.0)		12 (3.0)	
Overall accuracy, n/N (%)			383/401 (95.5)		380/401 (94.8)		388/401 (96.8)
**Misclassification analysis, n (%)**							
	Total errors	18 (4.5)		21 (5.2)		13 (3.2)	
	Missed findings	0 (0.0)		2 (0.5)		1 (0.2)	
	Downgraded cases	12 (3.0)		11 (2.7)		8 (2.0)	
	Upgraded cases	6 (1.5)		8 (2.0)		4 (1.0)	
New ESF (n=352, 87.8%), n (%)			363 (90.5)		360 (90.2)		352 (88.0)
Known ESF (n=49, 12.2%), n (%)			38 (9.5)		39 (9.8)		48 (12.0)
Correct specialty referral decision (n=401), n/N (%)			372/401 (92.8)		369/401 (92.0)		386/401 (96.3)

^a^ESF: extraspinal finding.

^b^C-RADS: CT Extracolonic Findings Reporting and Data System.

### Interobserver Agreement for C-RADS Classification

Gwet κ analyses demonstrated consistently high agreement across all comparisons (Table 3). Agreement between the human readers and the reference standard yielded kappas of 0.943 (95% CI 0.918-0.969) and 0.934 (95% CI 0.906-0.962) for readers 1 and 2, respectively (*P*<.001). Notably, the PP-LLM demonstrated the highest agreement with the reference standard, achieving a kappa of 0.959 (95% CI 0.937-0.981, *P*<.001), further supporting its potential to match or exceed expert-level performance in C-RADS classification of ESFs. All kappa values indicated almost perfect agreement (Gwet κ 0.81-1.00) according to the Landis and Koch scale.

**Table 3:** Gwet κ coefficients and 95% CIs comparing agreement between each human reader (readers 1 and 2), PP-LLM^a^, and the reference standard in C-RADS^b^ classification. Statistical significance for Gwet κ coefficients was evaluated against the null hypothesis of chance-level agreement (κ=0).

Comparison	Accuracy, %	Gwet κ (95% CI)	*P* value
Reader 1 vs reference	95.5	0.943 (0.918-0.969)	<.001
Reader 2 vs reference	94.8	0.934 (0.906-0.962)	<.001
PP-LLM vs reference	96.8	0.959 (0.937-0.981)	<.001
PP-LLM vs reader 1	N/A^c^	0.867 (0.845-0.890)	<.001
PP-LLM vs reader 2	N/A	0.859 (0.836-0.882)	<.001

^a^PP-LLM: privacy-preserving large language model.

^b^C-RADS: CT Extracolonic Findings Reporting and Data System.

^c^N/A: not applicable.

### PP-LLM Extraspinal Finding Identification, Classification, and Referral Requirement

The PP-LLM demonstrated strong overall performance (Table 2), correctly identifying 99.8% (400/401) of all ESFs, including all clinically urgent E4 findings such as aortic dissections. The model misclassified 2% (8/401) of cases into lower C-RADS categories, potentially underestimating findings that might benefit from follow-up, such as paranasal sinus mucosal thickening, and 1% (4/401) into higher categories, leading to potentially unnecessary referrals for benign findings like dependent lung changes. It also achieved 98.5% accuracy in distinguishing new from preexisting findings (Table 4) and 96.2% accuracy in assigning the correct specialty referral. Notably, the PP-LLM completed its end-to-end automated analysis in under 5 seconds per report. The reported processing time of under 5 seconds was measured from the point after the relevant EMR entries and MRI spine report had been provided as model input, to the generation of the PP-LLM recommendation. This timing therefore reflects model inference latency within the study workflow rather than the full end-to-end clinical workflow, which would additionally include document retrieval, preparation, clinician review, and downstream referral implementation.

When stratified by C-RADS category, the PP-LLM demonstrated consistently high performance across all metrics (Table 5). For E1/E2 findings—representing clinically insignificant observations—the model achieved an accuracy of 96.8%, a sensitivity of 94.7%, a precision of 91.8%, and an *F*_1_-score of 93.3%. For E3 findings, which are clinically indeterminate and may require further evaluation, performance was even stronger, with an accuracy of 97%, sensitivity of 97.3%, precision of 98.6%, and an *F*_1_-score of 97.9%. Notably, the model achieved perfect performance for all E4 findings, those deemed clinically significant and requiring urgent follow-up, demonstrating 100% accuracy, sensitivity, precision, and *F*_1_-score.

To compare classification performance between the PP-LLM and individual radiologists, the McNemar test (Table 4) was used to assess statistically significant differences in paired binary outcomes (ie, case-wise agreement with the reference standard: correct vs incorrect). There was no statistically significant difference in accuracy between PP-LLM and reader 1 (McNemar *P*=.36). The comparison between PP-LLM and reader 2 showed numerically higher accuracy with the model, although this did not reach statistical significance (McNemar *P*=.15).

The PP-LLM also demonstrated excellent performance in determining whether an ESF was new or preexisting, a crucial step in triaging for referral. Compared to two human readers (readers 1 and 2), the PP-LLM outperformed both across all evaluated metrics (Table 6). It achieved an overall accuracy of 98.5%, with near-perfect sensitivity (99.1%) and high specificity (93.9%). Precision and *F*_1_-score were similarly high, at 99.1% and 99.1%, respectively.

In contrast, readers 1 and 2 achieved lower overall accuracies of 95.3% and 94%, respectively. The PP-LLM demonstrated significantly higher accuracy in determining whether an ESF was new or preexisting compared with both reader 1 (McNemar *P*=.002) and reader 2 (McNemar *P*<.001). Readers 1 and 2 also demonstrated lower specificities of 69.4% and 67.3%, respectively, compared with the PP-LLM (McNemar *P*=.002 and *P*<.001, respectively; Table 6). These findings suggest that the PP-LLM may more effectively leverage EMR data to determine whether ESFs are new or preexisting, thereby reducing unnecessary false-positive referral triggers for stable or previously documented findings and improving prioritization of ESFs that may require specialist referral.

**Table 4:** Comparison of accuracy of C-RADS^a^ classification and accuracy and specificity of determining if ESF^b^ is new between PP-LLM^c^ and human readers (readers 1 and 2) using the McNemar test.

	PP-LLM vs reader 1	PP-LLM vs reader 2
Accuracy of C-RADS classification	96.8% vs 95.5% (McNemar *P*=.36)	96.8% vs 94.8% (McNemar *P*=.15)
Accuracy of determining if ESF is new	98.5% vs 95.3% (McNemar *P*=.002)	98.5% vs 94% (McNemar *P*<.001)
Specificity of determining if ESF is new	93.9% vs 69.4% (McNemar *P*=.002)	93.9% vs 67.3% (McNemar *P*<.001)

^a^C-RADS: CT Extracolonic Findings Reporting and Data System.

^b^ESF: extraspinal finding.

^c^PP-LLM: privacy-preserving large language model.

**Table 5:** PP-LLM^a^ performance on classifying ESF^b^ into various C-RADS^c^ categories. The single missed ESF by the PP-LLM belonged to the E1/E2 category and is included within the E1/E2 false negative count. False positive and false negative values are reported using a one-vs-rest multiclass classification framework and are therefore not directly additive across categories.

Category	True positive	True negative	False positive	False negative	Accuracy, %	Sensitivity, %	Precision, %	*F*_1_-score, %
E1/E2	90	298	8	5	96.8	94.7	91.8	93.3
E3	286	103	4	8	97.0	97.3	98.6	97.9
E4	12	389	0	0	100.0	100.0	100.0	100.0

^a^PP-LLM: privacy-preserving large language model.

^b^ESF: extraspinal finding.

^c^C-RADS: CT Extracolonic Findings Reporting and Data System.

**Table 6:** Performance comparison between the PP-LLM^a^ and human readers (readers 1 and 2) in determining whether ESFs^b^ are new. Classification performance was evaluated relative to the reference set of 401 ESFs. Missed ESFs were treated as false negatives for novelty determination because failure to detect an ESF precludes subsequent classification as new or preexisting.

Category	True positive	True negative	False positive	False negative	Accuracy, %	Sensitivity, %	Specificity, %	Precision, %	*F*_1_-score, %
Reader 1	348	34	15	4	95.3	98.9	69.4	95.9	97.3
Reader 2	344	33	16	8	94.0	97.7	67.3	95.6	96.6
PP-LLM	349	46	3	3	98.5	99.1	93.9	99.1	99.1

^a^PP-LLM: privacy-preserving large language model.

^b^ESFs: extraspinal findings.

## Discussion

### Principal Findings

In this retrospective study, we evaluated the feasibility and performance of a secure PP-LLM for automating the identification, classification, and referral triage of ESFs from MRI spine reports. Among 395 patients, 401 ESFs were detected, most commonly renal, gynecological, and thyroid abnormalities. Using a modified C-RADS system, nearly three-quarters of findings were classified as indeterminate (E3), while a small subset (3%, 12/401) were clinically significant and categorized as E4, requiring urgent follow-up. The PP-LLM achieved near-perfect accuracy in ESF detection (99.8%), correct specialty referral (96.3%), and determination of whether findings were new or preexisting (98.5%), outperforming experienced human readers. Notably, it achieved perfect performance in identifying all urgent E4 findings, such as aortic dissections, and completed analysis within 5 seconds per report. These results highlight the potential of a PP-LLM to improve efficiency, consistency, and safety in clinical workflows by integrating EMR context into structured triage decisions.

Traditional natural language processing (NLP) systems, such as CheXpert and DeepSpine, have demonstrated success in parsing radiology reports but rely on predefined rules and expert annotation, limiting adaptability across institutions [11-14]. Recent studies have shown that LLMs can overcome these constraints. For example, Hallinan et al [9,15] demonstrated that a secure institutional LLM could enhance MRI spine request forms and automate protocol selection, improving efficiency and standardization. Similarly, Vong et al [16] demonstrated the feasibility of using an LLM to automatically identify incidental hepatic steatosis from emergency department imaging reports.

More broadly, Park et al [5] showed that an open-source LLM could reliably label spinal MRI reports for AI training, Barash et al [17] demonstrated high concordance between ChatGPT-4 and the American College of Radiology (ACR) guidelines for emergency referrals, and Wiest et al [18] validated a privacy-preserving LLM for extracting cirrhosis data from free-text histories. Collectively, these studies illustrate the versatility of LLMs across radiology and health care administration. Our study builds on this foundation by directly applying a secure PP-LLM to referral triage, uniquely linking radiology reports with EMR context to support downstream patient management.

Beyond detection and classification, a key strength of the PP-LLM lies in distinguishing whether findings are new or preexisting, a determination that often drives referral decisions. In our study, the model achieved 98.5% accuracy with near-perfect sensitivity (99.1%) and high specificity (93.9%). By comparison, human readers showed lower accuracies (95.3% and 94%) and much lower specificities (69.4% and 67.3%). This specificity gap is clinically meaningful, as human readers tended to overcall findings as new. This may arise from cognitive fatigue with reviewing increasingly complex or fragmented EMR data that may span multiple subspecialty inputs across multiple time points [19,20]. Such overcalls can generate unnecessary referrals, adding to patient anxiety, health care costs, and subspecialty workload. The McNemar test confirmed that the improvements with the PP-LLM were statistically significant and showed that the model corrected human errors in most cases. These findings underscore the robustness of the PP-LLM in real-world clinical decision support, particularly in suppressing false-positive referrals without compromising safety.

While the PP-LLM performed strongly as a standalone tool, its optimal real-world deployment will likely involve augmented human oversight. In practice, this could take the form of an assisted review interface that highlights indeterminate or clinically significant findings (E3/E4) for expedited assessment, while benign or previously known findings (E1/E2) are automatically routed for routine follow-up. In addition, automated transmission of referral letters was not implemented in this study. Instead, outputs were intended to support clinician decision-making within a supervised workflow. This approach aligns with current best practices for deployment of clinical LLMs, where human verification remains essential to mitigate risks related to hallucinated outputs or inappropriate recommendations. Such a human-in-the-loop workflow would help ensure that critical findings are not overlooked [21], provide a safeguard against potential hallucinations, and maintain clinician accountability while still reducing administrative burden [22,23].

This approach aligns with the broader shift toward hybrid AI systems that enable seamless interfacing between radiology and multiple clinical specialties where automation complements rather than replaces expert judgment [24]. These studies underscore the adaptability of LLMs across clinician workflows and radiology integration, spanning protocol optimization, report standardization, and incidental findings management. Our work builds on this evidence by applying a privacy-preserving institutional LLM to a heterogeneous spectrum of ESFs, directly integrating radiology reports with EMR data to generate structured, actionable referral recommendations.

### Clinical Integration and Deployment Considerations

Beyond its technical accuracy, the true impact of the PP-LLM lies in its integration within the wider clinical ecosystem. Embedding the system into radiology reporting platforms and EMR interfaces could enable near–real-time triage of ESFs at the point of reporting. A unified clinical dashboard could then consolidate the detected ESFs, their corresponding C-RADS categories, and referral recommendations for clinician validation before report sign-off. Such integration would streamline multidisciplinary communication, reduce follow-up delays, and ensure consistent documentation of incidental findings across specialties.

Scalability and sustainability will depend on interoperability and local customization [25]. Institutions differ in their reporting styles, terminologies, and EMR configurations, all of which influence model generalizability. Continuous refinement through institution-specific fine-tuning and feedback loops where radiologists can flag errors or atypical cases for retraining will enhance robustness and adaptability [26]. Integration with PACS (picture archiving and communication system) or RIS (radiology information system) could further enable longitudinal tracking of ESFs, preventing duplicate referrals and ensuring that previously known findings are appropriately recognized.

In a proposed downstream clinical workflow, outputs from the PP-LLM may be used to support automated drafting of referral letters for new E3 and E4 extraspinal findings, subject to clinician review and approval. However, E4 findings represent critical diagnoses requiring immediate clinical escalation rather than passive referral-based follow-up. In future implementations, we propose incorporation of a tiered alert mechanism in which E4 classifications trigger real-time notification to the responsible clinician through existing hospital communication infrastructure, such as electronic urgent alert pathways, inpatient paging systems, or integration with institutional patient-safety escalation protocols. This would ensure that time-critical findings such as aortic dissection or other emergent pathologies are escalated appropriately without delay. In contrast, E3 findings, which are clinically significant but not immediately life-threatening, would continue to be managed through the standard automated referral-letter workflow under clinician oversight. Implementation of such a tiered escalation framework would require prospective institutional validation and workflow-integration testing prior to routine clinical deployment.

From an ethical and governance perspective, deployment must adhere to privacy frameworks such as the country’s data protection laws and institutional review board policies [18,27]. Privacy-preserving deployment architectures are particularly important in enabling safe clinical adoption of LLM-based decision-support systems, as deidentification alone is often insufficient for protecting patient confidentiality in longitudinal EMR datasets [28]. Institutionally governed inference workflows that restrict access to authorized users and maintain auditability of model interactions provide a practical pathway toward integrating structured and unstructured EMR data into automated clinical triage without requiring external data transfer [29]. This capability is especially relevant for incidental findings management, where determining whether findings are new or previously documented depends heavily on access to recent clinical history. In this context, deployment within secure hospital computing environments supports both data sovereignty and accountability, while incorporation of explainable AI features such as highlighting the textual cues underpinning referral decisions may further enhance transparency, strengthen clinician trust, and facilitate regulatory acceptance [30,31].

Ultimately, embedding the PP-LLM into routine clinical workflows has the potential to transform incidental findings management from a reactive, manual process into a proactive, data-driven system. By supporting radiologists and referring clinicians in the timely identification, classification, and referral of clinically significant ESFs, the PP-LLM could improve care continuity, reduce diagnostic delays, and alleviate administrative workload. Future prospective studies should evaluate its impact on referral completion rates, follow-up adherence, and patient outcomes, establishing its role as a scalable, safe, and clinically meaningful decision-support tool in routine practice.

### Limitations

This study has several limitations. First, this was a single-center retrospective study conducted at a tertiary academic institution, which may limit generalizability to centers with different reporting styles, patient populations, or EMR structures. However, the study included consecutive MRI spine reports across multiple spinal regions and clinical settings (inpatient and outpatient), and was reported by a department of independent radiologists, which partially mitigates selection bias and improves representativeness within our institutional workflow. Future multicenter studies across diverse reporting environments and health care systems are needed to validate generalizability [32]. Alternatively, repeating this single-center study on a temporally separated cohort could increase the dataset for robustness and validate reproducibility.

Second, the consensus reference standard was established through review by two experienced clinicians, with discrepancies adjudicated by a senior reviewer. As readers 1 and 2 contributed to the reference standard, strict statistical independence between individual reader performance and the ground truth cannot be fully assumed, introducing a potential source of incorporation bias [33]. Although adjudication by a senior reviewer improved the reliability of the consensus labels, comparisons between reader performance and the PP-LLM using the McNemar test should therefore be interpreted as exploratory rather than confirmatory inferential analyses. Future studies using externally adjudicated reference standards or multicenter validation cohorts would further strengthen the independence and generalizability of performance comparisons.

Third, while no major hallucinations or unsafe referral recommendations were observed in this study, LLM performance on rare or atypical but clinically relevant findings remains uncertain. This risk is slightly mitigated with prompt engineering to constrain and limit outputs. However, there may still be clinically significant findings that exist outside this reference standard. This can be further mitigated by proposing a human-in-the-loop deployment strategy in which referral recommendations are reviewed prior to implementation. Future prospective studies should evaluate model behavior in real-time clinical workflows and incorporate monitoring strategies to detect rare failure modes followed by adaptive strategies to refine the constraints and limiters in the prompt or the clinician-derived reference standard according to institutional practices.

Fourth, this study evaluated retrospective performance rather than real-world prospective deployment, and therefore the impact of the PP-LLM on referral completion rates, clinician workflow efficiency, and patient outcomes could not be directly assessed. However, the model demonstrated high agreement with expert reviewers and rapid processing time (<5 seconds per report), supporting its feasibility for clinical workflow integration. Prospective implementation studies are needed to evaluate downstream clinical impact and cost-effectiveness.

In addition, determination of whether ESFs were new or preexisting was based on a predefined 6-month EMR look-back window. While this timeframe was selected to provide a practical and reproducible assessment framework consistent across human reviewers and the PP-LLM, stable findings documented outside this window may have been classified as new observations. In real-world clinical workflows, such findings might already be known to treating teams through earlier documentation, and this constraint may therefore modestly inflate the apparent false-positive referral rate. Furthermore, the PP-LLM has not been evaluated in situations where clinical documentation spans an extended timeframe. A substantially larger volume of records may risk exceeding the PP-LLM optimal context window, potentially degrading analytical performance. Future prospective implementations could incorporate longer or adaptive EMR review windows to better reflect longitudinal documentation patterns across institutions.

Lastly, the modified C-RADS classification framework used in this study was adapted to reflect local referral practices at our institution, which may limit direct transferability to centers using different incidental finding management pathways. Nevertheless, the use of a structured and widely recognized classification framework should improve reproducibility and transparency of referral decision logic [34]. Future work should evaluate adaptation of the framework across institutions with different reporting conventions and subspecialty referral structures.

### Recommendations and Future Directions

Future work should validate the PP-LLM across diverse institutions and imaging modalities, ideally through prospective studies evaluating its impact on clinical workflows, referral timeliness, downstream patient outcomes, and health care resource utilization. In particular, the integration of the PP-LLM into spine clinic and radiology reporting pathways may help surgeons rapidly identify clinically relevant ESFs, distinguish new from previously known conditions, and prioritize appropriate subspecialty referrals earlier in the care pathway. Such workflow support has the potential to reduce administrative burden, improve care coordination between specialties, and minimize delays in follow-up of incidental but clinically significant findings.

Adaptive learning strategies, such as incorporating flagged discrepancies or rare findings into iterative model refinement pipelines, may further enhance reliability and institutional adaptability. In addition, institution-specific prompting strategies and domain-aligned fine-tuning may improve performance across heterogeneous reporting styles and EMR environments. With these developments, the PP-LLM has the potential to evolve into a scalable clinical decision-support tool that supports surgeons and referring clinicians in efficiently identifying, triaging, and coordinating interspecialty referrals arising from ESFs detected on MRI spine examinations.

### Conclusions

Our study demonstrates that a secure institutional PP-LLM can achieve high accuracy and efficiency in automating the identification, classification, and triage of ESFs from MRI spine reports. By operationalizing AI-driven automation at the clinician-radiology interface, this approach may reduce administrative burden, facilitate timely specialist referrals, and enhance overall patient care within existing multidisciplinary workflows. In future work, augmenting the PP-LLM with clinician oversight through clinical dashboards could help prioritize critical findings without sacrificing safety. Careful validation, continuous feedback, and seamless integration into clinical workflows will be essential to fully realize the benefits of this approach.

## Appendix

Multimedia Appendix 1 List of ESF classified according to organ/system involved based on the modified C-RADS. C-RADS: CT Extracolonic Findings Reporting and Data System; ESF: extraspinal finding.

Multimedia Appendix 2 Distribution of extraspinal findings according to the involved systems and diagnosis.

## Abbreviations

ACR: American College of Radiology
C-RADS: CT Extracolonic Findings Reporting and Data System
EMR: electronic medical record
ESF: extraspinal finding
LLM: large language model
MRI: magnetic resonance imaging
NLP: natural language processing
PACS: picture archiving and communication system
PP-LLM: privacy-preserving large language model
RAG: retrieval-augmented generation
RIS: radiology information system

## Data Availability

The datasets generated or analyzed during this study are available from the corresponding author on reasonable request.
