# Supplementary material for: Automating the Management of Extraspinal Findings in Magnetic Resonance Imaging Spine Studies Using a Privacy-Preserving Large Language Model: Retrospective Validation Study
Source: J Med Internet Res. 2026 Aug 26;28:e86251. doi: 10.2196/86251 (PMC13559147; doi:10.2196/86251)
Supplement: Supplementary file 1 [file jmir_v28i1e86251_app1.docx]

**Multimedia Appendix 1.** List of ESF classified according to organ/system involved based on the modified C-RADS.

| **C-RADS Extraspinal Findings Score** | **Definition** | **Example** |
| --- | --- | --- |
| **E1/E2** | No clinically important findings or benign findings that require no additional workup | Gastrointestinal:   - Diverticulosis - Cholelithiasis - Fatty liver - Splenic granulomas - Liver granulomas - Duodenal lipoma   Genitourinary:   - Non-obstructing renal calculi - Extra-renal pelvis. - Adrenal adenoma - Prostatomegaly - Nabothian cysts   Vascular:   - Vascular calcifications.   Musculoskeletal:   - Degeneration or osteoarthritis. - Subcutaneous oedema   Others:   - Mucus retention cyst |
| **E3** | Indeterminate, incompletely characterized findings, for which clinical correlation and further work-up and subspecialty referral could be performed if indicated. | Gastrointestinal:   - Simple or complex hepatic cyst - Mesenteric stranding and small lymph nodes. - Dilated common bile duct, with gallbladder present. - Hypoattenuating lesion in the pancreatic head, likely dilated common bile duct. - Prominent distal common bile duct after cholecystectomy. - Splenomegaly - High liver attenuation and enlarged inferior vena cava - Hyperattenuating lesion in gallbladder   Genitourinary:   - Simple or complicated renal cyst - Horse shoe kidney - Uterine Fibroid - Hydronephrosis - Homogeneously hyperattenuating renal lesion - Renal angiomyolipoma - Renal contour abnormality, cannot exclude exophytic mass - Medullary nephrocalcinosis - Adrenal nodule, soft-tissue attenuation - Pelvic mass - Cystic lesion adjacent to psoas muscle - Uterine adenomyosis. - Uterine cavity dilatation - Abdominopelvic fluid   Vascular:   - Ectatic abdominal aorta (2.6 – 2.9 cm)   Musculoskeletal:   - Lytic lesion in the ilium - Lipomatous lesion.   Others:   - Bulky thyroid gland or thyroid nodules. - Neck swelling. Salivary Gland Swelling. - Prominent lymph nodes. - Old infarcts - Bilateral apical lung scarring - Chronic sinusitis or mucosal thickening. - Mastoid effusion. - Hernias either containing fat or bowel with no complications. |
| **E4** | Potentially important findings requiring further work-up and early communication to the referring physician. | Gastrointestinal:   - Suspicious mass in the mesentery. - Suspicious hepatic lesion. - Suspicious bowel mass or lesion.   Genitourinary:   - Staghorn kidney calculus or other urolithiasis causing obstruction - Suspicious renal mass or lesion. - Bladder wall thickening or prostatic lesion   Musculoskeletal:   - Multiple bone lesions suggestive of metastasis or   multiple myeloma   - Unsuspected osteoporotic fracture   Vascular:   - Abdominal aortic aneurysm >3 cm   Others:   - Lung airspace consolidation suggesting pneumonia - Lung nodules - Bulky lymphadenopathy suspicious for malignancy - Obvious malignancy or lesions with high suspicion for malignancy |
