# Supplementary material for: Automating the Management of Extraspinal Findings in Magnetic Resonance Imaging Spine Studies Using a Privacy-Preserving Large Language Model: Retrospective Validation Study
Source: J Med Internet Res. 2026 Aug 26;28:e86251. doi: 10.2196/86251 (PMC13559147; doi:10.2196/86251)
Supplement: Supplementary file 2 [file jmir_v28i1e86251_app2.docx]

**Multimedia Appendix 2.** Distribution of extraspinal findings according to the involved systems and diagnosis.

| System | **Total** | | **ESF Diagnosis** |  |  |
| --- | --- | --- | --- | --- | --- |
|  | Number | Percentage (%) |  | Number | Referral to subspecialty (if required) |
| Genitourinary | 128 | 31.9 |  |  |  |
|  |  |  | Renal cysts/lesions | 75 | Urology |
|  |  |  | Duplex kidney | 3 |  |
|  |  |  | Prior nephrectomy | **2** |  |
|  |  |  | Atrophic kidneys | **2** |  |
|  |  |  | Prominent collecting system/hydronephrosis | **1** |  |
|  |  |  | Renal Angiomyolipoma | **3** |  |
|  |  |  | Urinary bladder wall thickening/debris | **2** |  |
|  |  |  | Catheterised urinary bladder | **1** |  |
|  |  |  | Prostatomegaly | **10** |  |
|  |  |  | **Others** | **29** |  |
| Gynaecological | 110 | 27.4 |  |  |  |
|  |  |  | Uterine Fibroids | 60 | Gynaecology |
|  |  |  | Uterine cyst | 2 |  |
|  |  |  | Uterine adenomyosis | 3 |  |
|  |  |  | Post-hysterectomy | 3 |  |
|  |  |  | Adnexal cyst | 20 |  |
|  |  |  | Nabothian cyst | 3 |  |
|  |  |  | Endometrial cavity fluid | 4 |  |
|  |  |  | Physiological fluid in the pelvis | 4 |  |
|  |  |  | Others | 11 |  |
| Endocrine-related | 48 | 12.0 |  |  |  |
|  |  |  | Thyroid nodules | 43 | Endocrinology |
|  |  |  | Adrenal nodules | 5 |  |
|  |  |  |  |  |  |
| **Gastrointestinal** | 36 | 9.0 |  |  |  |
|  |  |  | Hepatic cysts/lesions | 17 | Hepatology/Gastroenterology or General Surgery |
|  |  |  | Distended esophagus | 2 |  |
|  |  |  | Dilated biliary tree | 2 |  |
|  |  |  | Gallstones | 3 |  |
|  |  |  | Colonic diverticulosis | 3 |  |
|  |  |  | Bowel mural thickening | 1 |  |
|  |  |  | Inguinal Hernia | 3 |  |
|  |  |  | Abdominal Hernia | 2 |  |
|  |  |  |  |  |  |
|  |  |  | Others | 3 |  |
| **Vascular** | 7 | 1.7 |  |  |  |
|  |  |  | Dilated aorta/Aortic ectasia or aneurysm | 3 | Vascular Surgery |
|  |  |  | Aortic Dissection | 1 |  |
|  |  |  | Splenic varices | 1 |  |
|  |  |  | Ectatic/Hypoplastic vertebral artery | 1 |  |
|  |  |  | Cardiomegaly | 1 |  |
| **Musculoskeletal** | 5 | 1.2 |  |  |  |
|  |  |  | Lipomatous Lesion | 1 | Orthopaedic Surgery |
|  |  |  | Muscular Lesion | 1 |  |
|  |  |  | Arthropathy | 1 |  |
|  |  |  | Bone lesions | 1 |  |
|  |  |  | Hyperostosis frontalis | 1 |  |
|  |  |  |  |  |  |
| **Others** | 67 | 16.7 |  |  |  |
|  |  |  | Sinus mucus retention cyst | 22 | Otolaryngology |
|  |  |  | Sinusitis | 14 |  |
|  |  |  | Old infarcts | 2 | Neurology |
|  |  |  | Mastoid effusion | 2 | Otolaryngology |
|  |  |  | Lung scarring | 4 | Respiratory Medicine |
|  |  |  | Lung consolidation | 2 |  |
|  |  |  | Lung nodules/lesions | 3 |  |
|  |  |  | Pleural effusion | 5 |  |
|  |  |  | Parotid swelling | 2 | Otolaryngology |
|  |  |  | Lymph nodes enlargement | 3 | General Surgery |
|  |  |  | Skin lesions | 3 |  |
|  |  |  | Subcutaneous oedema | 2 |  |
|  |  |  | Sternotomy wires | 2 |  |
|  |  |  | Dental related | 1 |  |
